# 3′‐Sialyllactose protects against osteoarthritic development by facilitating cartilage homeostasis

**DOI:** 10.1111/jcmm.13292

**Published:** 2017-08-07

**Authors:** Jimin Jeon, Li‐Jung Kang, Kwang Min Lee, Chanmi Cho, Eun Kyung Song, Wook Kim, Tae Joo Park, Siyoung Yang

**Affiliations:** ^1^ Department of Biomedical Sciences Ajou University Graduate School of Medicine Suwon Korea; ^2^ Department of Pharmacology Ajou University School of Medicine Suwon Korea; ^3^ Korea Food Research Institute Seongnam‐si Gyeonggi‐do Korea; ^4^ School of Life Science Ulsan National Institute of Science and Technology Ulsan Korea; ^5^ Center for Genomic Integrity Institute for Basic Science Ulsan Korea; ^6^ Department of Molecular Science and Technology Ajou University Suwon Korea

**Keywords:** 3′‐sialyllactose, osteoarthritis, cartilage homeostasis

## Abstract

3′‐Sialyllactose has specific physiological functions in a variety of tissues; however, its effects on osteoarthritic development remain unknown. Here, we demonstrated the function of 3′‐sialyllactose on osteoarthritic cartilage destruction. *In vitro* and *ex vivo*, biochemical and histological analysis demonstrated that 3′‐sialyllactose was sufficient to restore the synthesis of Col2a1 and accumulation of sulphated proteoglycan, a critical factor for cartilage regeneration in osteoarthritic development, and blocked the expression of Mmp3, Mmp13 and Cox2 induced by IL‐1β, IL‐6, IL‐17 and TNF‐α, which mediates cartilage degradation. Further, reporter gene assays revealed that the activity of Sox9 as a transcription factor for Col2a1 expression was accelerated by 3′‐sialyllactose, whereas the direct binding of NF‐κB to the *Mmp3*,* Mmp13* and *Cox2* promoters was reduced by 3′‐sialyllactose in IL‐1β‐treated chondrocytes. Additionally, IL‐1β induction of Erk phosphorylation and IκB degradation, representing a critical signal pathway for osteoarthritic development, was totally blocked by 3′‐sialyllactose in a dose‐dependent manner. *In vivo*, 3′‐sialyllactose protected against osteoarthritic cartilage destruction in an osteoarthritis mouse model induced by destabilization of the medial meniscus, as demonstrated by histopathological analysis. Our results strongly suggest that 3′‐sialyllactose may ameliorate osteoarthritic cartilage destruction by cartilage regeneration *via* promoting Col2a1 production and may inhibit cartilage degradation and inflammation by suppressing Mmp3, Mmp13 and Cox2 expression. The effects of 3′‐sialyllactose could be attributed in part to its regulation of Sox9 or NF‐κB and inhibition of Erk phosphorylation and IκB degradation. Taken together, these effects indicate that 3′‐sialyllactose merits consideration as a natural therapeutic agent for protecting against osteoarthritis.

## Introduction

Osteoarthritis (OA) is a common cartilage degenerative disease that is primarily caused by the disruption of cartilage homeostasis 1, 2. Specifically, the underlying mechanism involves cartilage degradation through the induction of catabolic factors as well as down‐regulation of anabolic factors 3. Previous reports suggest that OA onset is associated with excessive production of interleukin‐1 beta (IL‐1β), which plays a key role in joint damage through the up‐regulation of metalloproteinases (Mmps) and cyclooxygenase2 (Cox2) activation and the down‐regulation of Col2a1 synthesis 4, 5. Moreover, IL‐6, IL‐17 and TNF‐α are also well characterized as pro‐inflammatory cytokines related to OA development 6, 7. Col2a1 constitutes a main component of cartilage extracellular matrix (ECM) proteins and is directly regulated by Sox9 8. Notably, loss of Col2a1 in chondrocytes induces chondrocyte dedifferentiation and leads to OA 8, 9. Mmp3 and Mmp13 represent critical factors in cartilage degeneration, as many catabolic pathways up‐regulate the activity of Mmp3 and Mmp13, which are highly effective in cleaving Col2a1, aggrecan and other ECM components 10. Although Cox2 is primarily involved in inflammation, the further increased activation of Mmps eventually leads to the induction of Col2a1 degradation during OA development 11. Consequently, the chondrocytes are not able to compensate for the depletion of Col2a1 during OA development by increased catabolic activity.

Molecular evidence clearly suggests that nuclear factor‐κB (NF‐κB) signalling plays a central role in Mmp3, Mmp13 and Cox2 expression and suppresses Sox9 expression, thus down‐regulating cartilage ECM proteins such as Col2a1 synthesis 8, 10, 12. IL‐1β has been shown to trigger activation of NF‐κB, p38, mitogen‐activated protein kinase (MAPK), c‐jun and NF‐κB signalling pathways, suggesting that these pathways are therefore involved in OA progression 6, 13. Thus, the activation of anabolic factor expression by Sox9 represents a good therapeutic target for protecting against osteoarthritis; however, currently developed therapeutic candidates are mostly focused on alleviating inflammation relief by Cox2 inhibition rather than facilitating cartilage regeneration by promoting ECM synthesis in chondrocytes.

Human milk oligosaccharides are considered to represent completely safe substances that have a variety of biological functions for skeletal growth and anti‐inflammatory effects 14; especially, sialyllactose, which is the core structure of sialyllactose human milk oligosaccharides, essentially constitutes sialic acid bound to a lactose molecule 15. 3′‐Sialyllactose, in which the N‐acetyl‐D‐neuraminic acid unit is connected to the galactose unit of lactose at the 3 position, has anti‐inflammatory properties and supports immune homeostasis 16. However, the function of 3′‐sialyllactose remains uncharacterized in the majority of degenerative diseases including OA.

Accordingly, the purpose of this study was to investigate the dual effect of 3′‐sialyllactose in regulating anabolic and catabolic processes in OA pathogenesis. In this study, we examined the role of 3′‐sialyllactose in the promotion of Col2a1 synthesis and inhibition of pro‐inflammatory cytokine (IL‐1β, IL‐6, IL‐17 and TNF‐α)‐induced Mmp3, Mmp13 and Cox2 expression by Sox9 and NF‐κB regulation, respectively. We further determined whether the dual function of 3′‐sialyllactose may have a particular therapeutic impact on OA development.

## Materials and methods

### Reagents and treatment

3′‐sialyllactose was purchased from GeneChem Inc. (Daejeon, South Korea). All pro‐inflammatory cytokine recombinant proteins (IL‐1β, IL‐6, IL‐17 and TNF‐α) were purchased from GenScript (Piscataway, NJ, USA). All reagents were dissolved in sterilized water and used in chondrocyte treatments. Chondrocytes were treated with IL‐1β (5 ng/ml), IL‐6 (50 ng/ml), IL‐17 (10 ng/ml) and TNF‐α (50 ng/ml) and cotreated with 3′‐sialyllactose at different concentrations (50, 100 and 250 μM) for 24 hrs prior to harvest.

### Culture of mouse articular chondrocyte and cartilage explants

Articular chondrocytes were isolated from femoral condyles and tibia plateaus of postnatal day 5 mice. Cartilage tissues were digested with 0.2% collagenase type II, as previously described 6. Cartilage explants were obtained from mouse knee joints and cultured in DMEM (Gibco‐BRL) containing 3′‐sialyllactose (100, 250 μM) with or without IL‐1β. The accumulation of sulphated proteoglycans was assessed by Alcian blue staining (1 volume of 0.3% Alcian blue 8GX in 70% ethanol, 1 volume of 100% acetic acid and 18 volumes of 100% ethanol), as described previously 17.

### Chondrocyte viability analysis

The chondrocytes were seeded in a 96‐well dish (9 × 10^3^ cells/well) for 48 hrs prior to treatments. 3′‐Sialyllactose was added at various concentration (10, 50, 100 and 250 μM) and incubated for 24 hrs in Dulbecco's modified Eagle medium (DMEM) without foetal bovine serum. Cytotoxicity was analysed with the EZ‐Cytox Cell viability assay kit (Dogen, Seoul, South Korea) following the manufacturer's manual. Briefly, 2‐(4‐iodophenyl)‐3‐(4‐nitrophenyl)‐5‐(2,4 disulfophenyl)‐2H‐tetrazolium (WST‐1) solution mixed with serum‐free DMEM (1:100, v/v) was added to cultured cells for 3 hrs and detected using a microplate reader VICTOR X3 (PerkinElmer, Waltham, MA, USA) at 450 nm.

### Reverse transcription–polymerase chain reaction (RT‐PCR) and quantitative (q)RT‐PCR

Total RNA was isolated from articular chondrocytes using TRIzol (Molecular Research Center Inc., Cincinnati, OH, USA). Isolated total RNA was reverse‐transcribed, and the resulting cDNA was amplified by PCR (Intron Biotechnology, Gyeonggi‐do, South Korea). PCR primers are summarized in Table S1. Transcript levels of target genes were quantified by qRT‐PCR using SYBR premix Ex Taq (TaKaRa Bio, Shiga, Japan). For each target gene, transcript levels were normalized to those of *Gapdh* and expressed as fold changes relative to the indicated controls.

### Western blotting and densitometry

Total proteins were extracted with lysis buffer (150 mM NaCl, 1% NP‐40, 50 mM Tris, 0.2% sodium dodecyl sulphate and 5 mM NaF) supplemented with a protease inhibitor and phosphatase inhibitor (Roche, Madison, WI, USA) cocktail inhibitor. Mmp3 and Mmp13 were detected after trichloroacetic acid precipitation as described 6. Each protein was visualized using the SuperSignal West Dura kit (Thermo Scientific, Waltham, MA, USA), and total Erk was used as a loading control. Western blot analysis was performed to detect protein levels using the following antibodies: mouse anti‐Mmp3 (ab52915; Abcam, Cambridge, UK), mouse anti‐Mmp13 (ab51072; Abcam), goat anti‐Cox2 (SC‐1745; Santa Cruz Biotechnology, Dallas, TX, USA), mouse anti‐Erk1/2 (610408; Becton Dickinson, New Jersey, New Jersey, NJ, USA USA), mouse anti‐pErk1/2 (#9101; Cell Signaling Technology, Boston, MA, USA), mouse anti‐IκB (9242; Cell Signaling Technology) and anti‐type II collagen (MAB8887; Millipore, Billerica, MA, USA). The relevant band intensities were quantified by densitometric analysis (AlphaEase FC 4.0; Alpha Innotech).

### Reporter gene assay, PGE2 assay and collagenase activity assay

The Sox9 or NF‐κB reporter gene constructs were transfected into mouse articular chondrocytes using LipofectAMINE Plus (Invitrogen, Carlsbad, CA, USA) as described 17, 18, 19. The transfected cells were cultured in complete medium for 24 hrs, and then the luciferase activity was determined using an assay kit (Promega, Madison, WI, USA) and subsequently normalized to β‐galactosidase activity. PGE_2_ production was assessed using a PGE_2_ immunoassay kit (R&D systems, Minneapolis, MN, USA)) as previously described 6. Primary chondrocytes were seeded in 96‐well plates (2 × 10^4^ cells/well). The levels of secreted and cellular PGE_2_ were quantified in total cell lysates. The total collagenase activity in the conditioned media of chondrocytes cultures was quantified with an EnzCheck Gelatinase/Collagenase Assay kit (Molecular Probes, Carlsbad, CA, USA). The collagenase activity was determined using a VICTOR X3 microplate reader (PerkinElmer, Waltham, MA, USA) at Ex/Em = 490/530 nm, according to the manufacturer's protocol.

### Destabilization of the medial meniscus (DMM)‐induced OA model and oral gavage

All animal experiments were approved by the Animal Care and Use Committee of the University of Ajou. For experimental OA models, 8‐week‐old male C57BL/6 mice were subjected to DMM surgery using a previously described protocol [20]. Mice knee joints were processed for histological analysis 10 weeks after surgery. Experimental OA mice also received gavage feeding of 3′‐sialyllactose (100 mg/Kg) or phosphate‐buffered saline (PBS) (controls) and process oral feeding on every other day during 4 weeks, and were killed 6 weeks after completion of the gavage feeding.

### Evaluation of cartilage destruction and immunohistochemistry

Cartilage destruction was assessed as previously described 6. Briefly, mouse knee joints were fixed in 4% paraformaldehyde, decalcified with 0.5 M EDTA (pH 8.0) for 2 weeks and embedded in paraffin. The paraffin blocks were sectioned at a thickness of 5 μm and were serial‐sectioned at 40‐μm intervals. Sections were deparaffinized in xylene and hydrated with graded ethanol. Cartilage destruction was detected by Safranin‐O staining and scored using the Osteoarthritis Research Society International (OARSI) grading system. Mmp3 (ab52915), Mmp13 (ab51072), Cox2 (SC‐1745), pErk ((#9101), IκB (9242), Sox9 (NBP2‐24659; Novus, Littleton, USA) and Col2a1 (MAB8887) were immunostained as previously described 17.

### Statistical analysis

Data were routinely presented as the means ± S.D. Significance was evaluated using Student's t‐test. Data quantified based on an ordinal grading system, such as OARSI grade, were analysed using nonparametric statistical methods. Significance was accepted at the 0.05 level of probability (*P *<* *0.05).

## Results

### 3′‐Sialyllactose promotes Col2a1 synthesis and accumulation of sulphated proteoglycan in articular chondrocytes and cartilage explants

We firstly determined whether 3′‐sialyllactose demonstrates cytotoxicity towards chondrocytes. Notably, treatment of the cells with different concentration of 3′‐sialyllactose for 36 hrs resulted in no observable cytotoxicity as determined by an MTT assay (Fig. S1). Thus, all subsequent *in vitro* analyses were performed with 3′‐sialyllactose from 0 to 250 μM.

**Figure 1:**
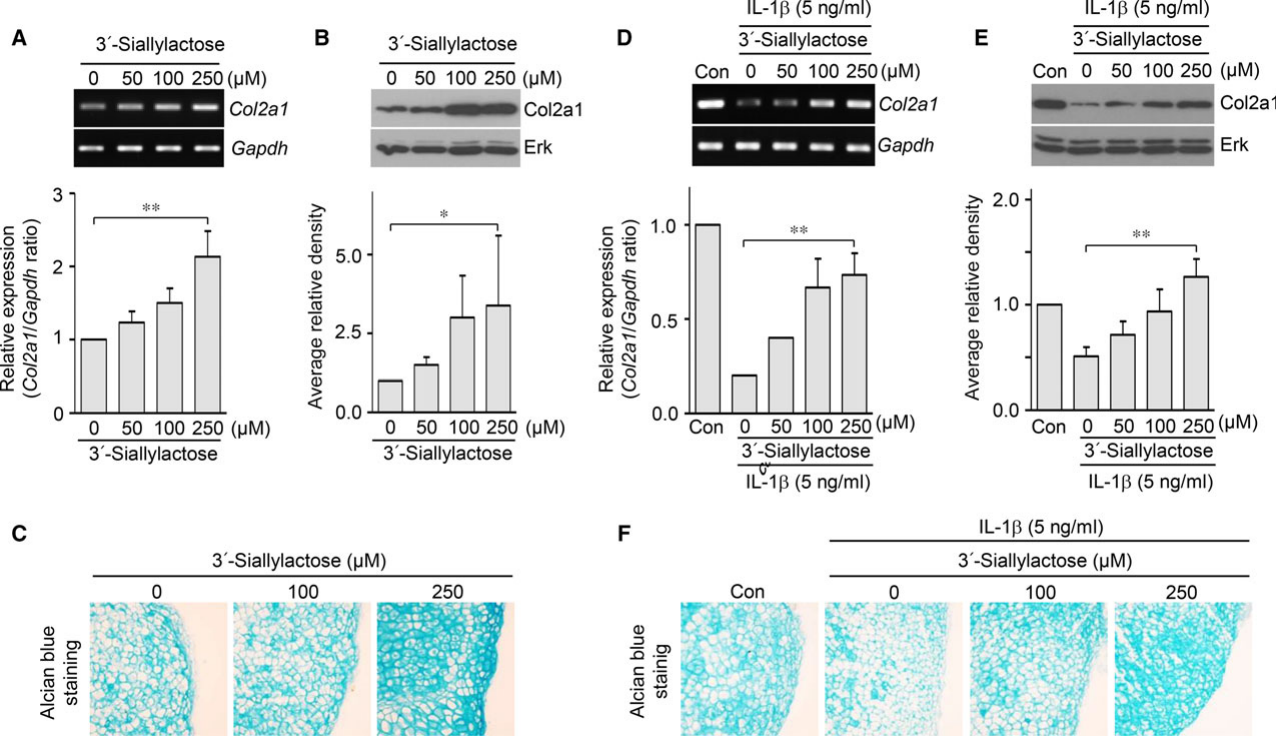
3′‐sialyllactose promotes and restores Col2a1 expression in articular chondrocytes. (**A**,** B**) Chondrocytes were treated with 3′‐sialyllactose in a dose‐dependent manner. Following 36 hrs of culture, Col2a1 was detected with RT‐PCR (**A**, upper panel), qRT‐PCR (**A**, lower panel), Western blot (**B**, upper panel) and densitometry (**B**, lower panel). (**C**) Articular cartilage explants were treated with 3′‐sialyllactose at the indicated dose. Following 72 hrs of explant culture, the accumulation of sulphated proteoglycans was assessed by Alcian blue staining. (**D**,** E**) Chondrocytes treated with IL‐1β (5 ng/ml) for 24 hrs were untreated or cotreated with 3′‐sialyllactose in a dose‐dependent manner. Col21 expression was determined by PCR (**D**, upper panel), qRT‐PCR (**D**, lower panel), Western blot (**E**, upper panel) and densitometry (**E**, lower panel). Gapdh and Erk were detected as loading controls. Values represent the means ± S.E.M. (*P <* *0.05, **P <* *0.0001). (**F**) Articular cartilage explants stimulated with IL‐1β were treated or not with 3′‐sialyllactose for 72 hrs. The accumulation of sulphated proteoglycans was determined by Alcian blue staining.

To investigate the function of 3′‐sialyllactose in chondrocytes, the gene and protein levels of Col2a1, a chondrocyte‐specific hallmark and the main component of ECM were determined by RT‐PCR, qRT‐PCR and Western blotting followed by densitometric analysis. As shown in Figure 1, Col2a1 transcript and protein levels were dramatically increased by 3′‐sialyllactose in a dose‐dependent manner (Fig. 1A and B). Additionally, 3′‐sialyllactose increased the accumulation of extracellular sulphated proteoglycans in cartilage explants (Fig. 1C). As these results suggest that 3′‐sialyllactose promotes Col2a1 synthesis and accumulation of extracellular sulphated proteoglycans in chondrocytes and cartilage explants, we next examined whether 3′‐sialyllactose restored Col2a1 expression during *in vitro* and *ex vivo* conditions that mimic OA such as IL‐1β treatment in chondrocytes and cartilage explants. IL‐1β constitutes a major pro‐inflammatory cytokine that is involved in cartilage destruction processes, such as inhibition of Col2a1 synthesis and activation of Mmps and Cox2 expression 21. Chondrocytes were treated with different concentrations of 3′‐sialyllactose in the absence or presence of IL‐1β. As expected, the expression of Col2a1 transcripts and protein was reduced in cells treated with IL‐1β; however, 3′‐sialyllactose restored Col2a1 expression in IL‐1β‐treated chondrocytes (Fig. 1D and E). Moreover, 3′‐sialyllactose restored the accumulation of extracellular sulphated proteoglycan in IL‐1β‐stimulated cartilage explants (Fig. 1F). These results suggest that 3′‐sialyllactose could promote and restore Col2a1 synthesis and accumulation of extracellular sulphated proteoglycan in a dose‐dependent manner during *in vitro* and *ex vivo* OA‐mimic conditions and may therefore possibly activate cartilage regeneration to facilitate OA development.

### 3′‐sialyllactose blocks IL‐1β‐induced Mmp3, Mmp13 and Cox2 expression in chondrocytes

**Figure 2:**
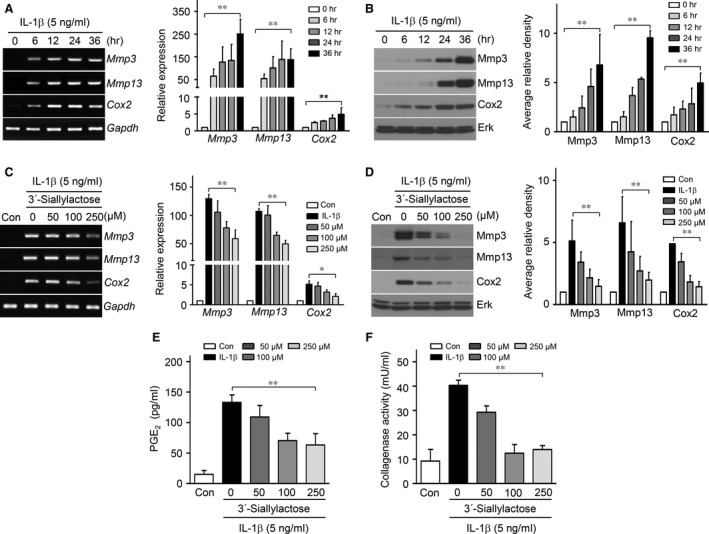
3′‐Sialyllactose inhibits IL‐1β‐induced Mmp3, Mmp13 and Cox2 expression in articular chondrocytes. (**A**,** B**) Chondrocytes were treated with IL‐1β (5 ng/ml) in a time‐dependent manner. Mmp3, Mmp13 and Cox2 expression levels were determined by PCR (**A**, left), qRT‐PCR (**A**, right), Western blot (**B**, left) and densitometry (**B**, right). (**C**,** D**) Chondrocytes treated with IL‐1β (5 ng/ml) were untreated or cotreated with or without 3′‐sialyllactose for 24 hrs in a dose‐dependent manner. The expression of Mmp3, Mmp13 and Cox2 was determined by PCR (**C**, left), qRT‐PCR (**C**, right), Western blot (**D**, left) and densitometry (**D**, right). Gapdh and Erk were detected as loading controls. PGE_2_ production (**E**) and collagenase activity (**F**) were detected in chondrocytes treated with IL‐1β and 3′‐sialyllactose. Values represent the means ± S.E.M. (*P <* *0.05, **P <* *0.0001).

Previous reports suggested that OA cartilage destruction is controlled not only by anabolic factor suppression but also by catabolic factor expression 22. We therefore next examined whether 3′‐sialyllactose blocked matrix degradation and inflammation activation through regulation of Mmps and Cox2 expression, respectively. The expression levels of Mmp3, Mmp13 and Cox2 were gradually increased by IL‐1β in a time‐dependent manner (Fig. 2A and B). To determine whether 3′‐sialyllactose blocked IL‐1β‐induced Mmp3, Mmp13 and Cox2 expression in chondrocytes, we treated cells with IL‐1β for 24 hrs in either the absence or presence of 3′‐sialyllactose. The expression of Mmp3, Mmp13 and Cox2 was dramatically decreased by 3′‐sialyllactose in a dose‐dependent manner (Fig. 2C and D). At the same time, PGE_2_ production (Fig. 2E) and collagenase activity (Fig. 2F) were dramatically reduced by 3′‐sialyllactose treatment in IL‐1β‐treated chondrocytes. Although tissue inhibitor of metalloproteinases (TIMPs) are known as natural inhibitors of Mmps 23, 3′‐sialyllactose did not affect TIMP expression (Fig. S2A and B). These results are consistent with the hypothesis that 3′‐sialyllactose protects against OA development by inhibiting PGE_2_ production and collagenase activity through Cox2 and Mmp (Mmp3 and Mmp13) expression, respectively, and by activating Col2a1 synthesis in chondrocytes.

### 3′‐Sialyllactose treatment reduced expression of Mmp3, Mmp13 and Cox2 induced by pro‐inflammatory cytokines (IL‐6, IL‐17 and TNF‐α) in chondrocytes

**Figure 3:**
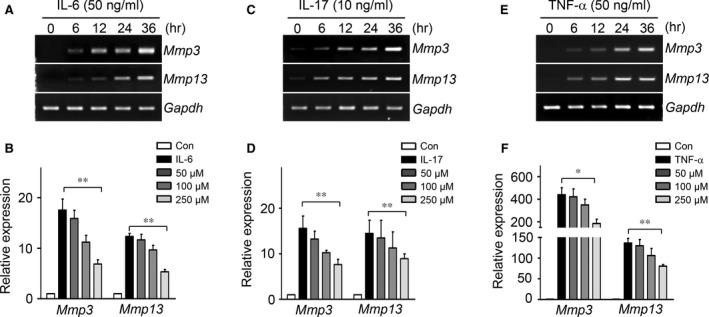
Mmp3 and Mmp13 expression induced by IL‐6, IL‐17 and TNF‐α was blocked by 3′‐sialyllactose in chondrocytes. Chondrocytes were treated with IL‐6 (50 ng/ml) (**A**), IL‐17 (10 ng/ml) (**C**) and TNF‐α (50 ng/ml) (**E**) in a time‐dependent manner. Chondrocytes treated with IL‐6 (**B**), IL‐17 (**D**) and TNF‐α (**F**) were cotreated with 3′‐sialyllactose for 24 hrs at the indicated concentration. The expression of Mmp3 and Mmp13 was determined by qRT‐PCR. Values represent the means ± S.E.M. (*P <* *0.05, **P <* *0.0001).

Although IL‐1β is one of the important OA‐related pro‐inflammatory cytokines, other pro‐inflammatory cytokines (IL‐6, IL‐17 and TNF‐α) have recently been found to play important roles in OA development 6, 7. To confirm the regulation of these pro‐inflammatory cytokines by 3′‐sialyllactose, we evaluated whether 3′‐sialyllactose inhibited the Mmp3 and Mmp13 expression induced by IL‐6, IL‐17 and TNF‐α in chondrocytes. As shown in Figure 3A, C and E, treatment with IL‐6, IL‐17 or TNF‐α could induce Mmp3 and Mmp13 expression, whereas the addition of 3′‐sialyllactose blocked the respective increases in Mmp3 and Mmp13 expression (Fig. 3B, D and F).

### Both activation of Sox9 and inhibition of NF‐κB are regulated by 3′‐sialyllactose in chondrocytes

**Figure 4:**
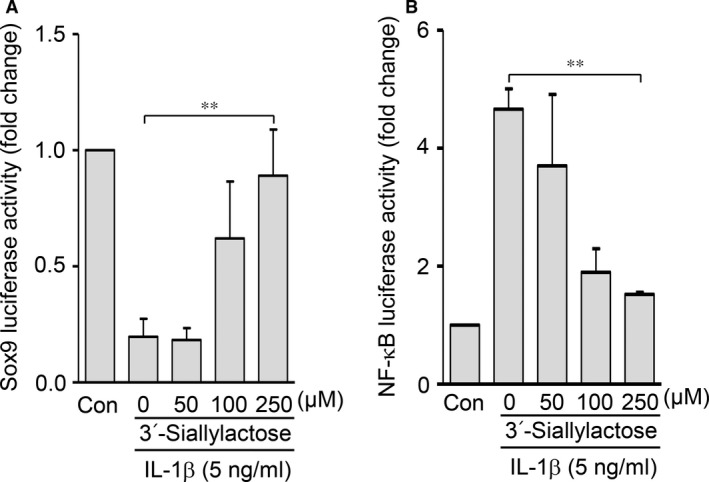
Activation of Sox9 and inhibition of NF‐κB are regulated by 3′‐sialyllactose. (**A** and **B**) Chondrocytes transfected with Sox9 or NF‐κB reporter gene constructs were treated with IL‐1β (5 ng/ml) in the absence or presence of 3′‐sialyllactose. Sox9 (**A**), and NF‐κB binding activity (**B**) was determined with a reporter gene assay. Data are presented as the results of mean values with standard deviations. Values represent the means ± S.E.M. (**P <* *0.0001).

To clarify the regulatory mechanisms of Col2a1 by 3′‐sialyllactose, we investigated whether Sox9, a master transcription factor for Col2a1 expression, was regulated by 3′‐sialyllactose in chondrocytes. A reporter gene construct containing the 48‐bp Sox9‐binding site in the first intron of human *COL2A1* 18 was employed to examine transcriptional activity. 3′‐sialyllactose restored the enhanced IL‐1β suppression of Sox9 transcriptional activity in a dose‐dependent manner (Fig. 4A). NF‐κB plays a central role in Mmps and Cox2 expression by directly binding their promoters (‐GGGRNYYCC; R: purine; Y: pyrimidine; and N: purine or pyrimidine) as well as by suppressing Sox9 expression, thus down‐regulating cartilage ECM proteins such as Col2a1 24. We therefore checked whether IL‐1β‐induced NF‐κB activity was decreased by 3′‐sialyllactose. Treatment of chondrocytes with IL‐1β led to activation of the NF‐κB transcription factor, whereas the IL‐1β‐induced activation of NF‐κB was blocked by the addition of 3′‐sialyllactose in a dose‐dependent manner as determined by transcriptional activation of the NF‐κB‐responsive promoter (Fig. 4B). These results clearly indicated that Col2a1 synthesis is enhanced by 3′‐sialyllactose induction of Sox9 transcriptional activity, whereas Mmp3, Mmp13 and Cox2 expression is inhibited by 3′‐sialyllactose suppression of NF‐κB activation in chondrocytes.

### Oral administration of 3′‐sialyllactose protects against cartilage destruction in the DMM‐induced OA model

**Figure 5:**
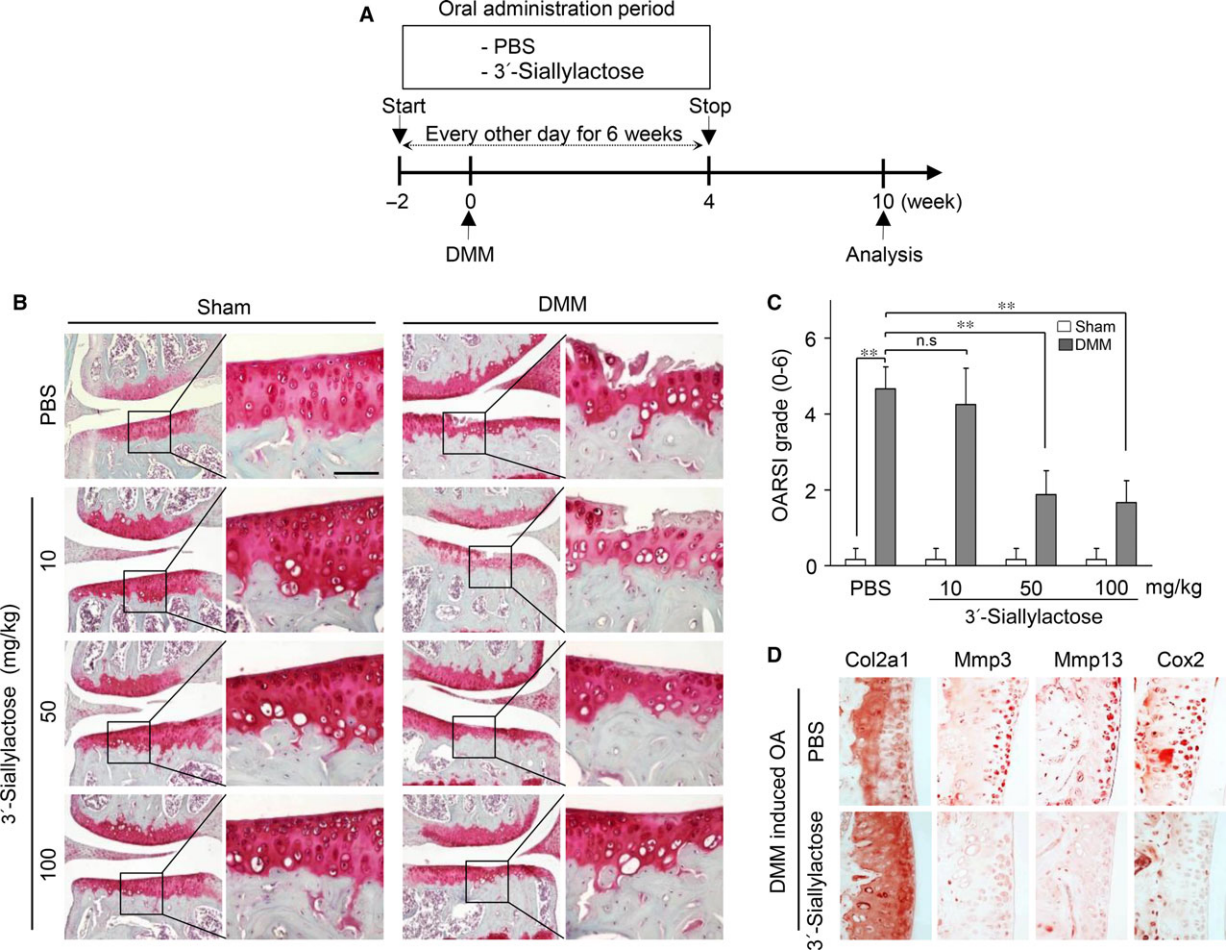
Oral administration of 3′‐sialyllactose protects against cartilage destruction in OA development. (**A**) Experimental scheme for analysis of the DMM‐induced OA model. (**B**) Mice with DMM were treated three times a week with 3′‐sialyllactose or PBS *via* oral gavage for 6 weeks. Cartilage destruction was detected by Safranin‐O staining. (**C**) Cartilage destruction was quantified by OARSI scores at 10 weeks following surgery, compared with DMM mice treated with PBS alone. Scale bar 50 μM. Values represent the means ± S.E.M.; **P <* *0.005. (**D**) Immunohistochemical staining of Col2a1, Mmp3, Mmp13 and Cox2 in DMM‐induced OA mice after oral administration of 3′‐sialyllactose.

To evaluate the role of 3′‐sialyllactose *in vivo*, we tested whether administration of 3′‐sialyllactose might protect against OA development in the DMM‐induced OA model. Mice were orally administered either control PBS or PBS containing 3′‐sialyllactose three times per week starting at 2 weeks prior to DMM and stopping 6 weeks before the end of the experiment (Fig. 5A). Compared to the PBS control group in DMM‐induced OA, oral administration of 3′‐sialyllactose resulted in dramatic protection against OA development as evident from significantly lower OARIS scores (Fig. 5B and C). Moreover, immunohistochemistry suggested that expression of Mmp3, Mmp13 and Cox2 was reduced in 3′‐sialyllactose‐administered DMM mice but not in PBS‐treated DMM mice (Fig. 5D). These data strongly suggest that 3′‐sialyllactose protects against cartilage destruction by catabolic factor expression in the DMM‐induced OA model.

### 3′‐Sialyllactose modulates Erk and NF‐κB signalling pathways for anabolic and catabolic factor expression

**Figure 6:**
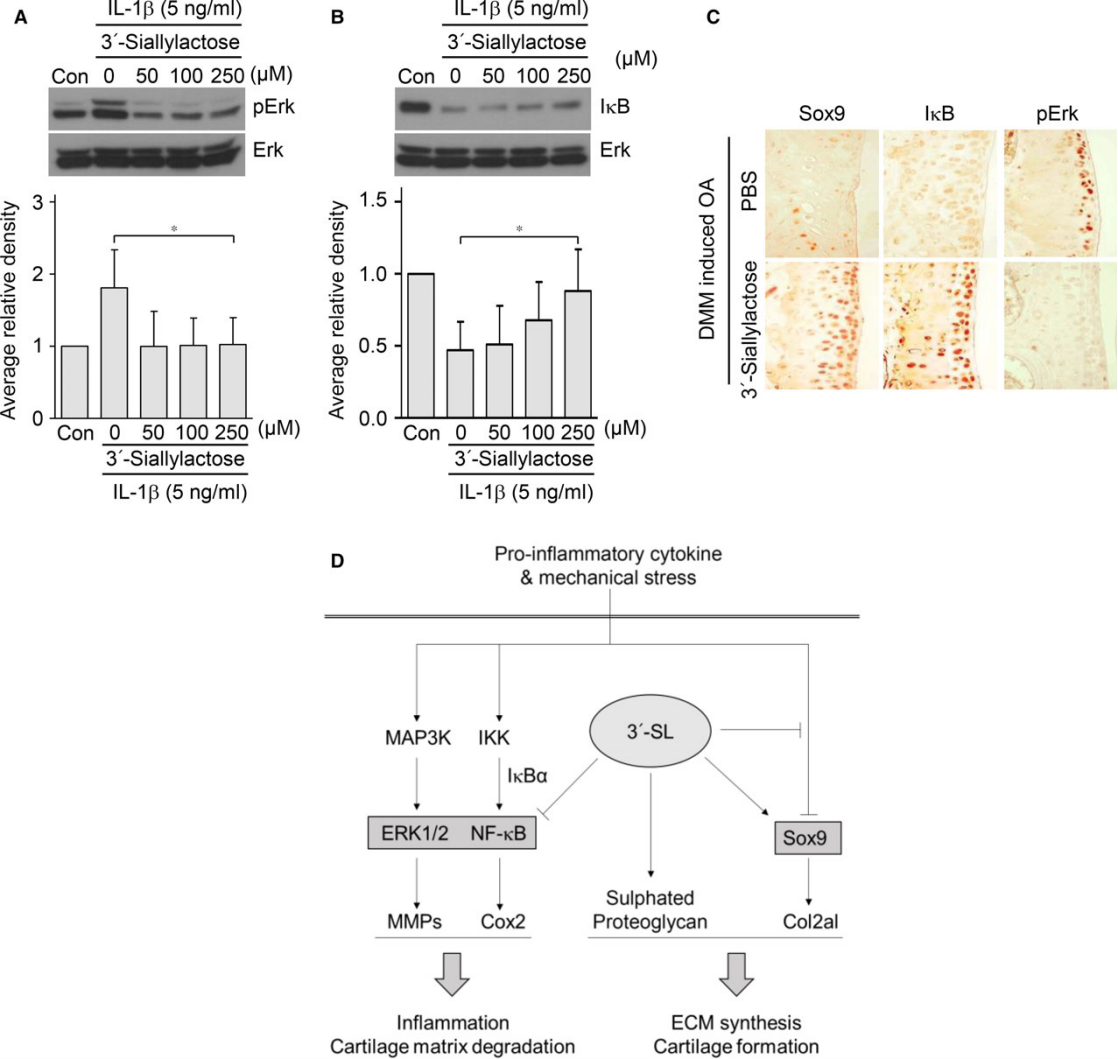
3′‐Sialyllactose regulates anabolic and catabolic factor expression *via* Erk and NF‐κB pathways. (**A**,** B**) Chondrocytes were pre‐treated with different concentrations of 3′‐sialyllactose for 24 hrs prior to treatment with IL‐1β (5 ng/ml) for 10 min. Erk phosphorylation was measured by Western blotting (**A**, upper panel) and densitometry (**A**, lower panel). The protein level of IκB was detected by Western blotting (**B**, upper panel) and densitometry (**B**, lower panel). Erk was detected as a loading control. (**C**) Immunohistochemical staining of Sox9, IκB and pErk in DMM‐induced OA mice with administration of PBS and 3′‐sialyllactose. (**D**) Schematic diagram of the signalling pathway leading to Sox9 activation and NF‐κB inhibition by 3′‐sialyllactose. Values represent the means ± S.E.M. *P <* *0.05.

NF‐κB and MAP kinase are activated by IL‐1β in chondrocytes 25. As NF‐κB and MAP kinase activity mediated by IL‐1β are involved in OA pathogenesis 6, 25, we further examined whether 3′‐sialyllactose might inhibit NF‐κB and MAP kinase signalling. Mouse articular chondrocytes were pre‐incubated for 12 hrs either in the absence or presence of 3′‐sialyllactose and exposed to IL‐1β (5 ng/ml) for 10 min. for the analysis of NF‐κB and MAP kinase signalling pathway activation. Among MAP kinase subtypes, the phosphorylation of ERK1/2 was markedly inhibited by 3′‐sialyllactose in IL‐1β‐treated chondrocytes (Fig. 6A), whereas the phosphorylation of JNK and p38 was not affected (data not shown). Further effect of 3′‐sialyllactose on the NF‐κB signalling pathway was suggested by the demonstration that degradation of IκB by IL‐1β was prevented by 3′‐sialyllactose (Fig. 6B). At the same time, Sox9 suppression, IκB degradation and levels of phosphorylated Erk (pErk) were reduced in 3′‐sialyllactose‐administered DMM‐induced OA mice but not in control mice (Fig. 6C). Taken together, our results clearly indicate that 3′‐sialyllactose up‐regulates cartilage regeneration and down‐regulates cartilage degradation through the inhibition of Erk and NF‐κB signalling activation in OA development (Fig. 6D).

## Discussion

In this study, we firstly found that 3′‐sialyllactose promotes cartilage regeneration and inhibits cartilage degradation by promoting Col2a1 synthesis and inhibiting Mmp3, Mmp13 and Cox2 expression, thereby protecting against OA development in a DMM‐induced OA model. Specifically, the underlying molecular mechanism of 3′‐sialyllactose is associated with Sox9 and NF‐κB transcriptional regulation *via* MAPK and NF‐κB signalling pathways both *in vitro* (chondrocytes) and *in vivo* (DMM‐induced OA mouse model).

Sialyllactose, an oligosaccharide, is commercially available and also occurs naturally in human milk 15. 3′‐Sialyllactose in particular exhibits anti‐inflammatory properties and supports immune homeostasis 16. 3′‐Sialyllactose as a prebiotic also influences colitis through the colonization of commensal bacteria 16, 26 and inhibits the attachment of pathogenic viruses and bacterial toxins to epithelial cells in the colon and lung 27, 28. However, as the effects of 3′‐sialyllactose in chondrocytes and OA development were largely unknown, in the current study we characterized these effects *in vitro* and in a DMM‐induced OA *in vivo* model.

Osteoarthritis represents a common cartilage degenerative disease that is driven by an imbalance between chondrocyte catabolism and anabolism 2. Specifically, during OA pathogenesis, cartilage ECM is depleted through the cessation of ECM protein synthesis and the activation of matrix degradation and inflammation 2, 29. Consequently, the chondrocytes are not able to compensate for the depletion of Col2a1, a main component of cartilage ECM, during OA development through increased catabolic activity. Notably, previous reports have suggested the role of IL‐1β in the molecular mechanism of OA development. IL‐1β constitutes the predominant pro‐inflammatory cytokine involved in the joint destruction associated with OA and has been widely used to mimic arthritis by its application to chondrocytes 30. IL‐1β plays a key role in joint damage through the up‐regulation of matrix degradation and inflammation as well as inhibition of ECM synthesis, which contribute to the destruction of arthritic cartilage 5. In the present study, IL‐1β significantly induced the expression of Mmp3, Mmp13 and Cox2, and suppressed Col2a1 synthesis in chondrocytes, thus mimicking the OA state as Mmp3 and Mmp13 are mainly involved in cleaving Col2a1 and other ECM proteins, thereby inducing cartilage degradation. Moreover, other pro‐inflammatory cytokines (IL‐6, IL‐17 and TNF‐ α) are known to induce Mmp3 and Mmp13 expression in chondrocytes 6, 7. Conversely, we found that 3′‐sialyllactose restored Col2a1 synthesis and accumulation of sulphated proteoglycan, whereas it inhibited Mmp3, Mmp13 and Cox2 expression under IL‐1β, IL‐6, IL‐17 and TNF‐α treatment.

Inflammation represents one of the causal factors of OA, and prostaglandin synthesis mediated by Cox2 expression is a major factor involved in cartilage inflammation. Furthermore, prostaglandin enhances Mmp synthesis and finally induces Col2a1 degradation during OA development 31. Accordingly, regulation of Cox2 expression is critical for developing an OA therapeutic target. The role of 3′‐sialyllactose in inflammation was thus further confirmed in the current study through the evaluation of Cox2 expression, PGE_2_ production and collagenase activity. In our experiments, Cox2 expression, PGE_2_ production and collagenase activity were dramatically decreased by 3′‐sialyllactose treatment in chondrocytes. As the DMM‐induced OA model is standard for studying mouse OA development among the various OA‐induced mouse models 20, we utilized this model to investigate whether 3′‐sialyllactose might protect against the development of OA *in vivo*. Our results strongly suggest that administration of 3′‐sialyllactose in DMM‐induced OA mice protects against cartilage destruction and reduces Mmp3, Mmp13 and Cox2 expression. Combined with the result that 3′‐sialyllactose also induced Col2a1 synthesis, these findings indicate a potential role for 3′‐sialyllactose in both activating cartilage regeneration and inhibiting cartilage degradation and inflammation during OA development.

At the transcriptional level, the transcription factor Sox9 directly promotes Col2a1 synthesis, whereas NF‐κB mainly binds to Mmp3, Mmp13 and Cox2 promoters at an NF‐κB binding site to regulate their expression 19, 20, 32. Our current results further indicated that 3′‐sialyllactose could restore Sox9 activity and suppress NF‐κB activity in IL‐1β‐stimulated chondrocytes. In this respect, the enhanced Col2a1 synthesis and suppression of Mmp3, Mmp13 and Cox2 expression by 3′‐sialyllactose may therefore underlie the regulation of Sox9 and NF‐κB by this compound.

Among various signalling pathways, it is well known that the MAPK and NF‐κB pathways are important in OA development through effects such as matrix degradation, inflammation and ECM synthesis 6, 13. In the current study, IL‐1β stimulation of chondrocytes resulted in the phosphorylation of MAPKs and inhibition of IκB degradation, confirming the role of IL‐1β in OA. We therefore assessed the inhibitory effect of 3′‐sialyllactose on the MAPK signalling pathway in chondrocytes and the DMM‐induced OA model. Our data strongly suggested that 3′‐sialyllactose inhibited the phosphorylation of Erk but not that of p38 and JNK (data not shown). As the regulation of catabolic factors is upstream of the NF‐κB signalling pathway, we observed that 3′‐sialyllactose affected the inhibition of IL‐1β‐induced IκB degradation in chondrocytes. Notably, the levels of inhibited Mmp3, Mmp13 and Cox2 and activated Col2a1 synthesis were additionally regulated upon inhibition of the Erk and NF‐κB signalling pathway 33. Therefore, 3′‐sialyllactose might also contribute to the regulation of MAP kinase and NF‐κB pathways.

In summary, our results collectively indicate that the activation of Col2a1 synthesis and inhibition of Mmp3 and Cox2 expression are regulated by 3′‐sialyllactose through its regulation of Sox9 and NF‐κB transcription *via* the MAPK and NF‐κB signalling pathways in articular chondrocytes. Therefore, it appears that 3′‐sialyllactose may completely block OA development in the DMM‐induced OA mouse model, thus meriting consideration as a therapeutic agent in OA.

## Conflict of interests

The authors declare that they have no competing interests.

## Supporting information


**Figure S1** Effect of 3′‐sialyllactose on chondrocyte viability.

**Figure S2** 3′‐sialyllactose does not promote Timp expression.

**Table S1** Primer sequence and PCR conditions.
