# Supplementary material for: 3′‐Sialyllactose protects against osteoarthritic development by facilitating cartilage homeostasis
Source: J Cell Mol Med. 2017 Aug 7;22(1):57–66. doi: 10.1111/jcmm.13292 (PMC5742729; doi:10.1111/jcmm.13292)
Supplement: Supplementary file 3 — Table S1 Primer sequence and PCR conditions. [file JCMM-22-57-s003.docx]

**Table S1**. Primer sequence and PCR conditions

| Gene | Origin | Strand | Sequence | Size  (bp) | AT^a^  (°C) |
| --- | --- | --- | --- | --- | --- |
| *Mmp3* | Mouse | ^b^S  ^c^As | 5'-CTGTGTGTGGTTGTGTGCTCATCCTAC-3'  5'-GGCAAATCCGGTGTATAATTCACAATC-3' | 350 | 58 |
| *Mmp13* | Mouse | S  As | 5'-TGATGGACCTTCTGGTCTTCTGGC-3'  5'-CATCCACATGGTTGGGAAGTTCTG-3' | 473 | 58 |
| *Cox-2* | Mouse | S  As | 5'-GGTCTGGTGCCTGGTCTGATGAT-3'  5'-GTCCTTTCAAGGAGAATGGTGC-3' | 724 | 65 |
| *Col2a1* | Mouse | S  As | 5'-CACACTGGTAAGTGGGGCAAGA-3'  5'-GGATTGTGTTGTTTCAGGGTTCG-3' | 173 | 58 |
| *Timp1* | Mouse | S  As | 5'-GCAACTCGGACCTGGTCATAA-3'  5'-CGGCCCGTGATGAGAAACT-3' | 226 | 60 |
| *Timp2* | Mouse | ^b^S  ^c^As | 5'-TCAGAGCCAAAGCAGTGAGC-3'  5'-CGGGTGTAGATAAACTCGATGTC-3' | 142 | 60 |
| *Timp3* | Mouse | S  As | 5'-CTTCTGCAACTCCGACATCGT-3'  5'-GGGGCATCTTACTGAAGCCTC-3' | 131 | 60 |
| *Gapdh* | Mouse | S  As | 5'-TCACTGCCACCCAGAAGAC-3'  5'-TGTAGGCCATGAGGTCCAC-3' | 450 | 55 |

^a^AT, annealing temperature; ^b^S, sense primer; ^c^As, antisense primer
